# If we build it they will come: targeting the immune response to breast cancer

**DOI:** 10.1038/s41523-019-0133-7

**Published:** 2019-10-29

**Authors:** Margaret E. Gatti-Mays, Justin M. Balko, Sofia R. Gameiro, Harry D. Bear, Sangeetha Prabhakaran, Jami Fukui, Mary L. Disis, Rita Nanda, James L. Gulley, Kevin Kalinsky, Houssein Abdul Sater, Joseph A. Sparano, David Cescon, David B. Page, Heather McArthur, Sylvia Adams, Elizabeth A. Mittendorf

**Affiliations:** 1 Laboratory of Tumor Immunology and Biology, National Cancer Institute, National Institutes of Health, Bethesda, MD USA; 2 Department of Medicine and Breast Cancer Research Program, Vanderbilt University Medical Center, Nashville, TN USA; 3 Division of Surgical Oncology and the Massey Cancer Center, Virginia Commonwealth University, Richmond, VA USA; 4 Division of Surgical Oncology, Department of Surgery, University of New Mexico; University of New Mexico Comprehensive Cancer Center, Albuquerque, NM USA; 5 University of Hawaii Cancer Center, Honolulu, HI USA; 6 University of Washington, Seattle, WA USA; 7 The University of Chicago, Chicago, IL USA; 8 Genitourinary Malignancies Branch, National Cancer Institute, National Institutes of Health, Bethesda, MD USA; 9 Columbia University Irving Medical Center, New York, NY USA; 10 Department of Medical Oncology, Montefiore Medical Center, Albert Einstein Cancer Center, Albert Einstein College of Medicine, Bronx, NY USA; 11 Division of Medical Oncology and Hematology, Department of Medicine, Princess Margaret Cancer Centre, University Health Network and University of Toronto, Toronto, ON Canada; 12 Providence Cancer Institute, Earle A. Chiles Research Institute, Portland, OR USA; 13 Cedars Sinai Medical Center, Los Angeles, CA USA; 14 Perlmutter Cancer Center, NYU School of Medicine, New York, NY USA; 15 Division of Breast Surgery, Department of Surgery, Brigham and Women’s Hospital, Boston, MA USA; 16 Breast Oncology Program, Dana-Farber/Brigham and Women’s Cancer Center, Boston, MA USA

**Keywords:** Breast cancer, Tumour immunology

## Abstract

Historically, breast cancer tumors have been considered immunologically quiescent, with the majority of tumors demonstrating low lymphocyte infiltration, low mutational burden, and modest objective response rates to anti-PD-1/PD-L1 monotherapy. Tumor and immunologic profiling has shed light on potential mechanisms of immune evasion in breast cancer, as well as unique aspects of the tumor microenvironment (TME). These include elements associated with antigen processing and presentation as well as immunosuppressive elements, which may be targeted therapeutically. Examples of such therapeutic strategies include efforts to (1) expand effector T-cells, natural killer (NK) cells and immunostimulatory dendritic cells (DCs), (2) improve antigen presentation, and (3) decrease inhibitory cytokines, tumor-associated M2 macrophages, regulatory T- and B-cells and myeloid derived suppressor cells (MDSCs). The goal of these approaches is to alter the TME, thereby making breast tumors more responsive to immunotherapy. In this review, we summarize key developments in our understanding of antitumor immunity in breast cancer, as well as emerging therapeutic modalities that may leverage that understanding to overcome immunologic resistance.

## Introduction

**Fig. 1:**
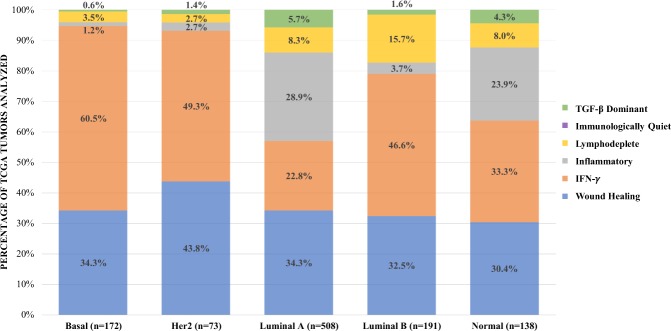
TCGA breast cancer subtype and percentage of corresponding immune subtype. (Generated from raw data in supplemental Fig. S1D in Thorsson et al.^1^)

Relative to melanoma, lung cancer, and other immunotherapy-responsive cancers, breast tumors have a lower tumor mutational burden, low tumor lymphocyte infiltration, and a low single-agent anti-PD-1/L1 response, leading some to characterize breast cancers as immunologically quiescent or “cold.” Recent evidence argues against this historical convention. The immune landscape of breast cancers is dynamic and heterogeneous, with significant variation observed across patients, subtypes and disease settings (early breast cancer v. metastatic). Using data on more than 10,000 samples across 33 different tumors available in The Cancer Genome Atlas database, Thorsson et al.^1^ identified six distinct immune subtypes including wound healing, interferon (IFN)-γ dominant, inflammatory, lymphocyte depleted, immunologically quiet and transforming growth factor beta (TGF-β) dominant. Among breast cancers (*n* = 944), the most common immunogenomic subtypes identified were IFN-γ dominant, followed by wound healing, and inflammatory (Fig. 1).^1^ Sixty percent of basal-like breast cancers were of the IFN-γ dominant subtype and a little less than half of HER2-enriched (HER2+) and luminal B breast cancers were of the IFN-γ dominant subtype. The IFN-γ dominant subtype has the highest CD8^+^ T-cell (cytotoxic T lymphocytes; CTLs) and M1 macrophage (pro-immune) density, as well as a high degree of T-cell receptor (TCR) diversity. A higher lymphocyte expression signature, defined as a higher number of unique TCR clonotypes, higher cytokines made by activated Th1 and Th17 cells and more M1 macrophages, improved survival in the IFN-γ dominant subtype as well as in the wound healing subtype.

Importantly, no breast cancers in this analysis were identified as immunologically quiet.^1^ This highlights the importance of other elements of the tumor microenvironment (TME; composed of diverse immune cells, cytokines and stroma) in modulating the immune response against breast tumors. Improved understanding of the complexity of host-tumor interactions in the TME has led to the possibility of targeting elements within the microenvironment to expand clinical responses to immune therapies.^1^

## Adaptive immune responses

The adaptive immune system detects foreign cells through recognition of non-self (such as viral or tumor proteins) or through inappropriate expression of self or mutated antigens. After successful clearance of these antigens, a pool of memory T-cells are retained indefinitely and provide lasting immunity. However, as is the case with inflammation resulting from chronic infection, a system of checks and balances exists within the normal functioning of adaptive immunity in order to limit sustained tissue injury and generate tolerance to persistent antigens. With a malignancy, multiple steps along this elimination process can fail. Examples of such defects include failure to harbor, express, or present immunogenic peptides, the increased secretion of immunosuppressive cytokines (e.g. TGF-β, interleukin [IL]-8, IL-6, IL-10), the activation of regulatory T-cells (Tregs) or the upregulation of immune checkpoints or their ligands on T-cells and stromal/tumor cells, respectively.

**Fig. 2:**
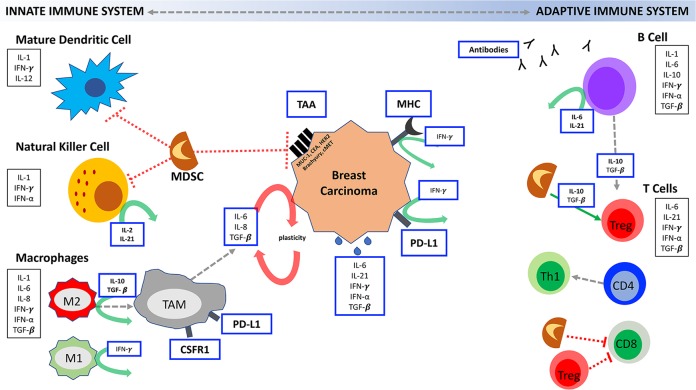
Interaction between the innate and adaptive immune system is vital for immune recognition and elimination of breast tumors. Activation of antigen presenting cells, natural killer cells, macrophages and engagement of T-cells and B-cells through the release of host-derived cytokines plays a central role to tumor destruction. To evade the immune system, tumors release cytokines and skew the tumor microenvironment to a more immunosuppressive environment through inhibiting CD8^+^ T cells, NK cells, dendritic cell maturation and through increasing Tregs and tumor associated macrophages (TAMs). Tumors also reduce antigen presentation of tumor-associated antigens (TAAs) on the tumor surface, and major histocompatibility complex (MHC) expression and alter the antigen presentation machinery (effector cells) to further reduce immune recognition. As this complex web of interactions demonstrates, there are multiple opportunities for the use of immunotherapeutic drug combinations in breast cancer. Figure Key: Blue boxes = targets for immunotherapy drugs; Black boxes = cytokines released by immune cells or tumor; green arrows = activation; red dotted line = inhibiton

Tumoral immune cell infiltration is predictive and prognostic in some breast cancer subtypes. The importance of the composition of the infiltrating immune cells (T-cell, B-cell, natural killer [NK] cell, etc.) is still being determined; however, breast tumors with higher tumor infiltrating lymphocytes (TILs) are more responsive to treatments (e.g., immunotherapy, chemotherapy, radiation) than those with low TILs. In tumors with few or no immune cells in the TME, various methods can be utilized to help shift the balance and attract immune cells. Methods to help mobilize professional antigen presenting cells (APCs) (e.g. dendritic cells [DCs] and macrophages) or effector cells (e.g. NK cells or CD8^+^ T-cells) include therapeutic vaccines, monoclonal antibodies, and cytokines (Fig. 2). Once these cells are in the TME, T-cells and B-cells can be better engaged through the use of agents like immune checkpoint blockade (ICBs).

### Tumor infiltrating lymphocytes

**Fig. 3:**
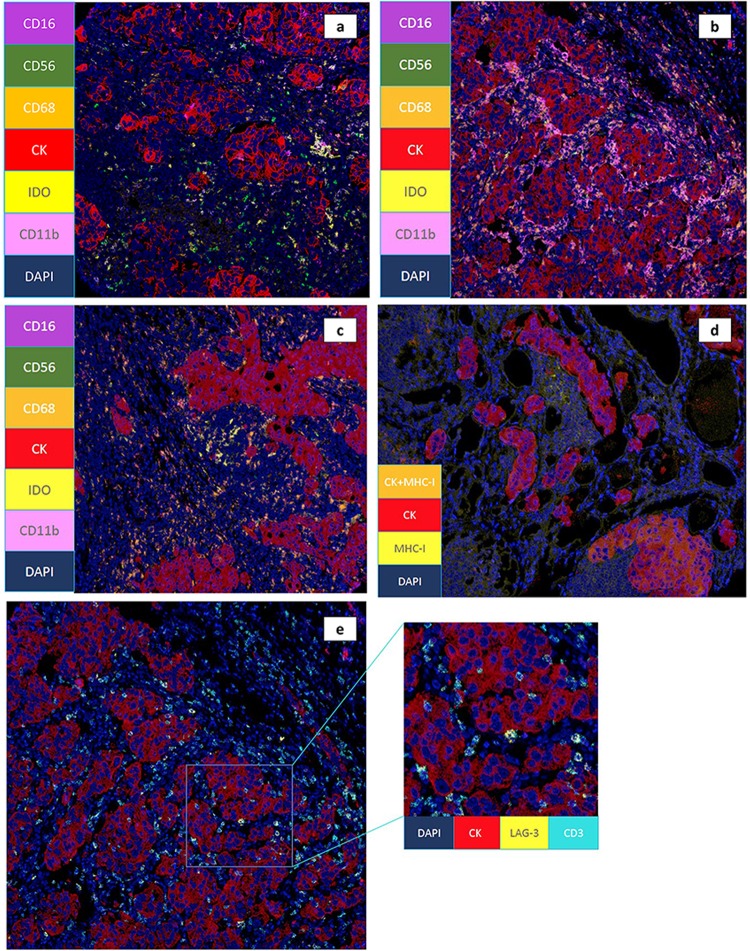
Innate immune cell infiltrates in breast cancer microenvironment. Triple negative breast tumors (CK) with natural killer cells (NK; CD16, CD56), myeloid cells (CD16, CD11b), and macrophage (CD68) infiltration in addition to expression of the immune inhibitory enzyme indoleamine 2,3-dioxygenase (IDO). **a** Breast tumor (CK) with a predominance of NK cells along with focal IDO expression by macrophages and myeloid cells. **b** Breast tumor (CK) with a myeloid cell predominance (CD16, CD11b). **c** Breast tumor (CK) with NK cells (CD16, CD56), myeloid (CD16, CD11b), macrophage (CD68) and tumor (CK) in addition to IDO expression. **d** Breast tumor (CK) with expression of major histocompatibility 1 (MHC-1). The areas of tumor expressing both CK (red) and MHC-1 (yellow) markers result in an orange hue while areas of tumor that have lost MHC-1 expression are of red color only. **e** Breast tumor (CK) with expression of CD3^+^ T-cells expressing lymphocyte-activation gene 3 (LAG3) in contact with tumor cell nest. All images were created by Houssein Abdul Sater using MIBITracker open resource software (https://mibi-share.ionpath.com) by IONPath.^5^ FFPE breast cancer tissue was stained and imaged using multiplexed ion beam imaging by time-of-flight (MIBI-TOF)

Di Paola et al.^2^ reported more than 40 years ago that lymphocyte infiltration in breast cancers and signs of immune activation in the regional lymph nodes are highly predictive of better clinical outcomes. Some degree of TIL infiltration is found in most breast cancers, but the proportions vary greatly among breast cancer subtypes.^3^ Standardized methodology for assessing TIL in hematoxylin and eosin (H&E) sections is validated.^4^ Newer techniques, such as multiplexed immunofluorescent (IF) staining, allow for assessment of multiple cell types and markers on a single histologic section (Fig. 3^5^) and may provide a better understanding of the complexity of the immune microenvironment in cancers.

TILs are classified as stromal or intratumoral and are graded visually on H&E sections using a continuous scale.^4^ The degree and type of lymphocytic infiltrate is prognostic in the neoadjuvant,^6^ adjuvant^7^ and metastatic^8^ settings for triple-negative breast cancer (TNBC) and HER2+ breast cancer as well as predictive of a higher likelihood of a pathologic complete response (pCR) with neoadjuvant chemotherapy.^9^

Single-cell approaches have not been thoroughly explored, but preliminary reports show a high degree of B-cells and T-cells (as well as macrophages, which are not included in standard TILs scoring metrics) in the TME of primary breast cancers.^10^ TILs isolated from breast tumors are mainly composed of memory CD4^+^ and CD8^+^ T-cells. The significance of infiltrating FOXP3^+^ CD4^+^ T-cells (also known as Tregs) is somewhat paradoxical; it is likely that the ratio to CD8^+^ is more important (Fig. 3). The presence of Tregs is associated with a poor prognosis and recurrence risk, especially for hormone receptor-positive (HR+) breast cancer.^11^

### Immune checkpoint blockade – PD-1/PD-L1, LAG-3, TIGIT

Single-agent ICBs have produced durable responses in a small proportion of breast cancer patients.^12,13^ This may be augmented by the addition of cytotoxic chemotherapy (*see companion manuscript*; Page DB et al.), as evidenced by the recent FDA approval of atezolizumab with nab-paclitaxel for first line, metastatic, PD-L1+ TNBC using the Ventana assay (SP142).^14^

Among targetable checkpoints, both PD-1 and lymphocyte-activation gene 3 (LAG-3) positive TILs have been noted in a subset of patients with highly infiltrated tumors, but their presence did not appear to impact prognosis beyond TILs expression alone.^15,16^ Nonetheless, both PD-1 and LAG-3 are well-established immunosuppressive molecules that can be targeted by clinically available inhibitors, and may help define a population of patients who could benefit from ICB combinations. Recent studies have also intriguingly identified high expression of B7-H4, a PD-L1 family member, on tumor cells of poorly immune infiltrated breast tumors.^17^ However, the receptor for B7-H4 (analogous to PD-1 and presumably present on T-cells, which are suppressed in the presence of B7-H4^18^) has not yet been defined, despite the clinical development of B7-H4-blocking antibodies. Finally, the checkpoint TIGIT has been noted to be enriched in ductal carcinoma in situ compared to invasive ductal cancers, which were more enriched for PD-L1, but the implications of this finding are currently unknown.^19^ There are ongoing, phase I clinical trials involving anti-PD-1/L1 ICBs combined with LAG-3 (NCT03250832; Table 1), B7-H4 (NCT03514121), or TIGIT (NCT03628677) blockade in solid tumors, with planned expansion into breast cancer.

**Table 1:** Ongoing combination immunotherapy clinical trials in breast cancer

Trial target	Trial information (trial name)	Trial description	Primary outcome(s)	Open date (estimated completion)
Immune checkpoints	NCT03250832	Status: recruiting Setting: solid tumors including BC (*n* = 260) Treatments: TSR-033 (anti-LAG3) +/− anti-PD-1	• Safety (TRAEs) • ORR	Aug 1, 2017 (May 2021)
	NCT03514121	Status: recruiting Setting: solid tumors including BC (*n* = 278) Treatments: pembrolizumab + FPA150 (anti-B7-H4)	• Safety (MTD, RP2D, TRAEs)	Mar 27, 2018 (Jan 2024)
	NCT03628677	Status: recruiting Setting: solid tumors including BC (n = 242) Treatments: AB122(anti-PD-1) + AB154 (anti-TIGIT)	• Safety (TRAEs)	Aug 21, 2018 (Feb 2020)
Synthetic chimeric antigen receptors (CARs)	NCT01837602	Status: completed, results not published. Setting: advanced TNBC Treatments: cMET RNA CAR T cells	• Safety (SAEs)	Apr 23, 2013 (Oct 2018)
	NCT02792114	Setting: recruiting Setting: metastatic HER2+ BC (*n* = 36) Treatments: mesothelin-targeted T-cells, metronomic cyclophosphamide	• Safety (MTD)	Jun 2016 (Jun 2020)
B-cells	NCT02403271	Status: completed, results not published Setting: solid tumors with TNBC and HER2+ BC (*n* = 124) Treatments: ibrutinib + durvalumab	• Safety (AEs, RP2D) • ORR	Mar 2015 (Jan 2019)
Inflammatory cytokines	NCT03135171	Status: recruiting Setting: metastatic HER+ BC (*n* = 20) Treatments: toclizumab (anti-IL-6) + herceptin/pertuzumab	• Safety (RP2D)	May 22, 2017 (Aug 2020)
	NCT02370238 (FRIDA)	Status: active, not recruiting Setting: metastatic TNBC (*n* = 156) Treatments: reparixin (anti-IL-8) + paclitaxel	• PFS	Jun 2015 (Feb 2019)
	NCT02672475	Status: recruiting Setting: metastatic AR negative TNBC (*n* = 29) Treatments: galunisertib + paclitaxel	• Safety (AEs, MTD)	Mar 2016 (Jun 2021)
	NCT03524170	Status: recruiting Setting: metastatic HR+ /HER2-BC (*n* = 20) Treatments: M7824 + RT	• Safety (RP2D, tolerability)	Apr 30, 2018 (Sep 2020)
	NCT03579472	Status: recruiting Setting: metastatic TNBC (*n* = 20) Treatments: M7824 + eribulin	• Safety (RP2D, AEs)	May 30, 2018 (Oct 2020)
	NCT03620201	Status: recruiting Setting: neoadjuvant HER2+ BC (*n* = 20) Treatments: M7824 neoadjuvant	• Change in TILs	Aug 3, 2018 (Dec 2019)
	NCT03742349	Status: recruiting Setting: metastatic TNBC (*n* = 220) Treatments: spartalizumab + LAG525 (anti-LAG3) + variety of IO agents including canakinumab (anti-IL-1β)	Safety (AEs, SAEs, DLTs, dose reduction/interruptions)	Jan 31, 2019 (Oct 2020)
	NCT02983045 (PIVOT-2)	Status: recruiting Setting: solid tumors including TNBC (*n* = 480) Treatments: NKTR-214 (anti-CD122) + nivolumab +/− ipilimumab	• Safety and tolerability (TRAEs, SAEs, DLTs, discontinuation) • ORR	Oct 2016 (Jun 2021)
	NCT03435640 (REVEAL)	Status: recruiting Setting: solid tumors including TNBC (*n* = 393) Treatments: bempegaldesleukin (IL-2) + NKTR-262 (TLR 7/8)+/− nivolumab	• Safety (AEs, SAEs) • Tolerability (DLTs, TRAEs, SAEs, AEs) • ORR	Mar 15, 2018 (Dec 2022)
	NCT03328026	Status: recruiting Setting: breast (*n* = 40) Treatments: SV-BR-1-GM (vaccine with IFN-α and post-treatment metronomic cyclophosphamide) + pembrolizumab	• Safety (AEs, SAEs)	Mar 16, 2018 (Dec 2020)
	NCT02675439	Status: recruiting Setting: solid tumors including BC (*n* = 75) Treatments: MIW815 + /− ipilimumab	• Safety (TRAEs, DLTs, RP2D)	Mar 2016 (Dec 2020)
	NCT03172936	Status: recruiting Setting: solid tumors including BC (n = 150) Treatments: MIW815 + spartalizumab	• Safety (DLTs)	Sep 8, 2017 (Dec 2020)
Toll-like receptors (TLRs)	NCT01042379 (I-SPY 2)	Status: recruiting Setting: neoadjuvant BC (*n* = 1920) Treatments: SD-101 + Pembrolizumab	• pCR • RCB • RFS • OS • Safety (AEs, SAEs)	Mar 2010 (Dec 2020)
Natural killer cells	NCT02627274	Status: recruiting Setting: solid tumors including HER2+ BC (*n* = 205) Treatments: RO6874281 +/− trastuzumab	• Safety (DLT, MTD, RP2D) • PKs	Dec 7, 2015 (May 2020)
	NCT03319459	Status: recruiting Setting: solid tumors including HER2+ BC (n = 100) Treatments: FATE-NK100 (donor derived NK cell product) + trastuzumab	• Safety (DLT)	Jan 18, 2018 (Oct 2022)
Myeloid-derived suppressor cells	NCT02393794	Status: recruiting Setting: metastatic TNBC (n = 54) Treatments: romidepsin + cisplatin +/− nivolumab	• Safety (RP2D) • ORR	Mar 19, 2015 (Jul 2020)
	NCT02637531	Status: recruiting Setting: solid tumors including TNBC (*n* = 220) Treatments: duvelisib (IPI-549) + nivolumab	• Safety (AEs, DLTs)	Dec 2015 (Apr 2020)
Tumor-associated macrophages	NCT02265536	Status: completed, results not published. Setting: metastatic BC (*n* = 36) Treatments: LY3022855 (anti-CSF1R)	• Changes in peripheral immune cell subsets • Changes in serum cytokines	Oct 2014 (Feb 2018)
	NCT01596751	Status: active, not recruiting Setting: metastatic TNBC (*n* = 68) Treatments: eribulin + PLX3397 (anti-CSF1/CSF1R)	• Safety (MTD) • PFS at 12 weeks	May 11, 2012 (May 2018)
	NCT02435680	Status: active, not recruiting Setting: metastatic TNBC (n = 50) Treatments: MSC110 (anti-CSF1) + carboplatin + gemcitabine	• PFS	May 6, 2015 *(Jun 2019)*
	NCT02824575	Status: recruiting Setting: metastatic HER2-BC (n = 60) Treatments: rebastinib + paclitaxel or eribulin	• Safety (RP2D)	Jul 2016 (Jul 2020)

Of note, data presented in this table was confirmed from www.clinicaltrials.gov on August 13, 2019

*AEs* adverse events, *AR* androgen receptor, *BC* breast cancer, *BOR* best overall response, *CBR* clinical benefit rate, *DLT* dose limiting toxicity, *DOR* duration of response, *HR+* hormone receptor positive, *HER2* human epidermal growth factor 2, *IO* immuno-oncology, *MTD* maximum tolerated dose, *ORR* objective response rate, *OS* overall survival, *pCR* pathologic complete response, *PD* pharmacodynamics, *PFS* progression free survival, *PKs* pharmacokinetics, *RCB* residual cancer burden, *RFS* recurrence free survival, *RP2D* recommended phase 2 dose, *RT* radiation therapy, *SAEs* severe adverse events, *TIL* tumor infiltrating lymphocyte, *TNBC* triple negative breast cancer, *TRAEs* treatment related adverse events

### Adoptive T-cell therapy

Adoptive immunotherapy, the administration of immune effector T-cells, has been assessed as an anticancer strategy for several decades.^20^ Adoptive therapy approaches are classified according to whether natural or genetically modified cell products are used. The first approach involves the harvest, expansion and re-infusion of autologous TILs. This method was pioneered at the National Cancer Institute in the 1980s, and recently resulted in durable remission of metastatic HR+ breast cancer in a patient treated with autologous TILs enriched for T-cells reactive against autologous neoantigens and administered following lymphodepleting chemotherapy and in combination with pembrolizumab.^21^ Further validation, as well as characterization of the contribution of pembrolizumab, is awaited.

The second major approach involves genetically engineered therapeutic T-cell products that have been retargeted through the transfer of either a tumor specific TCR, or a synthetic chimeric antigen receptor (CAR) derived from an antibody’s antigen binding domains.^22^ TCRs recognize major histocompatibility complex (MHC) presented peptides (derived from intracellular proteins), while CAR-T-cells directly recognize surface expressed proteins, without the need for antigen presentation (often downregulated in cancer). Although these strategies can be transformative in the context of obligate, lineage-dependent targets (e.g., CD19 in B-cells), the identification of universally expressed tumor specific targets in solid tumors is a challenge, and reactivity against normal tissues is a source of potentially serious toxicity, as observed in a case of lethal toxicity (attributed to lung epithelial expression) following treatment with HER2-directed CAR-T.^23^ Multiple targets have been evaluated in preclinical studies, including cMET (expressed in HER2+ and TNBC) and mesothelin (expressed in TNBC) which are now advancing to the clinic (NCT01837602 and NCT02792114, respectively).

### B-Cells

B-cells make up a significant portion of TILs in many cancers, including breast cancer.^24^ Their ability to produce antibodies, present antigens, secrete cytokines and interact with immune cells allows for diverse functions that modulate the TME and immune responses towards a pro-tumor or antitumor response.^25^ Mouse models of solid tumor development show deficient tumorigenesis in the absence of B-cells.^26^ Conversely, CD20^+^ B-cell TILs in breast cancer are associated with improved survival and lower relapse rates.^27^

There is emerging evidence for a regulatory B-cell subset (Breg), with a distinct function in attenuating antitumor immune responses. Bregs suppress immune responses via the release of anti-inflammatory mediators, such as IL-10, IL-35, and TGF-β, which trigger T-cell conversion to Tregs.^25^ In the 4T1 mouse breast cancer model, the primary effect of tumor-evoked Bregs within lung metastasis is the induction of TGF-β-dependent conversion of resting CD4^+^ T-cells to FOXP3^+^ Tregs.^28^ Also in the 4T1 model, inactivation of Stat3 with resveratrol decreased metastases through inactivation of tumor-evoked Breg cells.^29^ In breast cancer patients, metastasis-free survival was significantly shorter for patients with the coexistence of Tregs and Bregs in TIL aggregates compared to Tregs alone, suggesting their interdependence in the development of breast cancer metastasis.^30^

Ibrutinib irreversibly binds to Bruton’s tyrosine kinase and inhibits B-cell development. Ibrutinib also promotes T-cell cytotoxicity and an M1 macrophage phenotype^31^ leading to potential therapeutic uses in solid tumors. Ibrutinib is being evaluated in combination with the anti-PD-L1 antibody durvalumab in solid tumors, including breast cancer (NCT02403271).

### Bridging the adaptive and innate immune systems

Cytokines and APCs link the innate and adaptive immune systems. Cytokines mediate this connection, while antigen processing and presenting cells functionally bridge the innate and adaptive immune systems.

### Cytokines

Many cytokines function to recruit specific cell types to an inflammatory microenvironment. Cytokines impact metastatic potential, tumor progression, and angiogenesis. Cytokines vary among different breast cancer stages (early stage vs metastatic).^32^ Aberrant overexpression of a range of proinflammatory cytokines by breast tumors has been reported.^33–35^ Cytokines may augment or inhibit the host immune response,^32^ and many cytokines are pleiotropic with both tumor-promoting and antitumor effects (Table 2). Various cytokines that enhance antitumor activity have been evaluated in the preclinical and clinical settings. While some benefit has been documented, there is often a narrow therapeutic window with systemic administration, making these agents difficult to use. Subcutaneous and intratumoral administration help avoid some of the systemic effects. However, regardless of delivery method, one of the main limitations of cytokines is that activity depends on the presence of an existing host immune response.^32^

**Table 2:** Cytokine effects on the breast tumor microenvironment

Cytokine	Source	Cytokine effect on tumor	Clinical agents in development for use in breast cancer
IL-1	B-cells Mature DCs NK cells Macrophages	Pro-Tumor • Promotes tumor invasion • Promotes inflammation	IL-1 Receptor Antagonist • Anakinra (NCT01802970) • IL-1 Neutralizing mAB • Canakinumab (NCT03742349)
IL-2	Activated T-cells NK cells DCs Macrophages	Antitumor • Activates NK cells • Activates CD8^+^ T-cells	IL-2 Analogues • Aldesleukin (NCT00006228) • Bempegaldesleukin (NCT02983045; NCT03435640)
IL-6	B-cells TAMs Breast tumor	Pro-Tumor • Treatment resistance • Increased tumor plasticity • Promotes metastasis	IL-6 Antagonist • Tocilizumab (NCT03135171)
IL-8	TAMs Breast tumor	Pro-Tumor • Increased tumor plasticity • Increased MDSCs	IL-8 Neutralizing mAB • Humax IL-8 (NCT02536469) • IL-8 Receptor Antagonist • SX-682 (protocol in development) • Reparaixin (NCT02370238)
IL-10	TAMs B-cells	Antitumor (pleiotropic) • Pro-Tumor • Promote Tregs • Promote M2 phenotype	IL-10 Antagonist • Pegilodecakin (NCT02009449)
IL-21	CD4^+^ T-cells Breast tumor	Antitumor • Stimulates NK cells • Stimulates CTLs	IL-21 Analogues • Recombinant IL-21 (no further clinical development at this time)
IFN	DCs NK cells Macrophages B-cells T-cells Breast tumor	Antitumor • Enhanced antigen presentation • Increased CTL-killing	IFN Analogues • Human-leukocyte IFN (NCT03328026) IFN Stimulating Agents • MIW815 (NCT02675439)
TGF-β	TAM Breast tumor Macrophages	Antitumor (Early Stage Disease; pleiotropic) Pro-Tumor (Late Stage Disease) • Promotes M2 • Therapeutic resistance • Immune suppression	TGF-β Receptor 1 Inhibitor • Galunisertib (NCT02538417; NCT02672475) TGF-β Receptor 2 Inhibitor • M7824 (NCT03579472; NCT-3524170; NCT03620201)

Analogues increase the activity (or concentration) of a specific cytokine within the TME

Antagonists and neutralizing antibodies decrease the activity (or concentration) of active cytokine within the TME

*CTL* cytotoxic T lymphocyte, *DC* dendritic cell, *IFN* interferon, *IL* interleukin, *mAB* monoclonal antibody, *MDSC* myeloid derived suppressor cells, *NK* natural killer, *TAM* tumor associated macrophages, *TGF-β* transforming growth factor beta

Many breast cancers exhibit an inflammatory signature within the TME, which is associated with poor clinical outcomes.^35,36^ Decreased antitumor responses are due to (1) the release of immunosuppressive cytokines (e.g., IL-4, IL10, IL-13, IL-33, IL-35, IL-37, and TGF-β), (2) the recruitment of cells with immunosuppressive effects like Tregs, myeloid derived suppressor cells (MDSCs) or (3) the lack of recruitment of cells with immunomodulatory effects like CD8^+^ T-cells, NK cells and DCs.

Tumor plasticity (also known as epithelial-to-mesenchymal transition) is driven by an autocrine loop involving multiple proinflammatory cytokines, including IL-6, IL-8, and TGF-β.^33,37^ In addition to release from effector cells (i.e., T-cells, B-cells, NK cells), IL-6, IL-8, and TGF-β are also produced and released by tumor cells and tumor-associated macrophages (TAMs). Overexpression of IL-6 and IL-8 across multiple tumor types, including breast cancer, is associated with tumor progression, metastasis, therapy resistance and/or poor clinical outcomes.^33,37–39^ High circulating levels of IL-6 are associated with advanced disease, higher risk of recurrence and aggressive phenotypes in breast cancer.^39^ Preclinical studies have demonstrated that tumor secretion of IL-6 is related to treatment resistance, including tamoxifen resistance in luminal breast cancer cell lines and trastuzumab resistance in HER2-overexpressing breast cancer cell lines.^39^ Administration of tocilizumab, an IL-6 antagonist, reduced the cancer stem cell population in mouse xenografts and reduced tumor growth and metastasis.^39^ There are limited clinical data on IL-6 blockade in breast cancer, with only one ongoing trial using tocilizumab in combination with trastuzumab and pertuzumab in patients with trastuzumab-resistant, metastatic HER2+ breast cancer (NCT03135171).^38,39^ Preclinical neutralization of IL-8 with HuMax-IL8 has been shown to reduce plasticity of claudin-low TNBC in vivo, decrease tumor recruitment of MDSCs, and augment tumor sensitivity to NK- and T-cell-mediated lysis.^40^ There are multiple anti-IL-8 or IL-8 receptor (CXCR1 and CXCR2) blocking agents being evaluated in the preclinical and clinical settings. Anti-IL8 agents being evaluated in the clinical setting for breast cancer include SX-682 (trial not yet open) and reparixin (NCT02370238).

In early stages of malignant transformation, TGF-β is a tumor suppressor. In later stages of tumor development, it promotes tumor aggressiveness and metastasis. Loss of TGF-β inhibition and increased TGF-β signaling have been associated with cancer progression, stemness, therapeutic resistance as well as immune suppression.^41^ Several TGF-β targeting agents are being examined in breast cancer. Galunisertib, a TGFβRI/ALK5 inhibitor, is being evaluated in combination with radiotherapy (NCT02538471; *trial terminated due to slow accrual*) or with paclitaxel (NCT02672475). A phase I study of M7824, a first-in-class bifunctional antibody targeting TGF-βRII and PD-L1, in heavily pretreated, advanced solid carcinoma patients showed a manageable safety profile and signs of efficacy.^42^ There are multiple ongoing clinical trials involving M7824 in combination with standard of care in breast cancer (with radiation, NCT03524170; with eribulin, NCT03579472; in a neoadjuvant window study, NCT03620201).

Cytokines can drive both innate and adaptive immune responses, but they also play a role in Treg activation, tumor invasion and angiogenesis. For example, IL-1 is released by effector cells and plays a central role in immune and inflammatory responses in breast cancer, specifically tumor invasion.^34^ Preclinical studies of treatment with the IL-1 receptor antagonist anakinra promoted significant breast tumor control in mice. A pilot study evaluated anakinra followed by standard chemotherapy in women with HER2-negative metastatic breast cancer and demonstrated a sustained decrease in the expression of multiple genes for Toll-like receptor (TLR) and IL-1β families, while increasing the expression of NK and CD8^+^ T-cells genes that were associated with tumor lysis.^36^ The anti-IL-1β neutralizing monoclonal antibody canakinumab is currently being evaluated in combination with various immunotherapy agents in TNBC (NCT03742349). Other therapeutic approaches, including IL-1α blockade and a therapeutic vaccine targeting IL-1β are in clinical development.^43^

IL-21 is naturally produced by CD4^+^ T-cells and is a potent regulator of NK cells and CD8^+^ T-cells. Recent reports in murine models suggest that IL-21 may enhance trastuzumab efficacy in metastatic HER2+ breast cancer through modulatory effects on NK cells and CD8^+^ T-cells, Tregs, and macrophages.^44^ Due to enhanced NK-cell directed lysis of tumor cells bound by antibodies (also known as cell-mediated antibody-dependent cellular cytotoxicity; ADCC), IL-21 was combined with cetuximab in a phase I trial for patients with metastatic colorectal cancer, but the trial was stopped prematurely due to toxicity.^45^ Despite initial promise in clinical trials, dose-limiting toxicities and lack of consistent clinical activity have hampered clinical development of IL-21.^32^ There are no published clinical reports of IL-21 agents in breast cancer.

IL-2 enhances NK cell and CD8^+^ T-cell function.^34^ Recombinant IL-2 (aldesleukin) is an FDA-approved therapy for metastatic melanoma and renal cell carcinoma. Results from a pilot phase I study in metastatic HER2+ breast cancer indicated that IL-2 combined with trastuzumab was well tolerated and provided clinical benefit.^46^ However, no responses were observed in the subsequent phase II study for patients who progressed on trastuzumab.^47^ Preclinical studies with PEGylated IL-2 (bempegaldesleukin or NKTR-214) have demonstrated superior antitumor activity and immune activation profile relative to aldesleukin.^48^ A phase I trial examining PEGylated IL-2 as a single agent in metastatic solid tumors showed evidence of antitumor activity and a favorable safety profile.^49^ This agent is now being examined in combination with multiple immuno-oncology agents (including nivolumab) in TNBC (NCT02983045; PIVOT). An ongoing phase Ib study (NCT03435640; REVEAL trial) is examining the combination of PEGylated IL-2 with a TLR7/8 agonist in heavily pretreated patients with metastatic solid tumors, with a planned trial expansion in TNBC.

IL-10 is a pleiotropic cytokine that exhibits both tumor-promoting and inhibitory properties. IL-10 is expressed in patients with breast cancer and has been associated with poor prognosis.^35,50^ A recent preclinical study combining PEGylated human IL-10 (AM0010 or pegilodecakin) with docetaxel in 4T1 (TNBC) mouse models demonstrated synergy, with complete responses in 75% of mice.^51^ However, given its paradoxical role in tumor development and uncertainty about how best to shift activities to tumor inhibition (rather than tumor promotion), few IL-10 targeting agents are in clinical development. One trial evaluated PEGylated human IL-10 (AM0010) in solid tumors (*n* = 51) and showed an acceptable toxicity profile with evidence of systemic immune activation and antitumor activity (27% overall response rate).^52^ To the best of our knowledge, there are no plans for further evaluation of PEGylated human IL-10 in breast cancer patients.

Clinical trials with type I and II IFNs in solid malignancies have had variable success, and are associated with moderate-to-severe toxicities. Non-inflamed human tumors lack type I IFNs, which are important in both innate and adaptive immune-mediated tumor eradication.^53^ IFN-α and IFN-β enhance tumor antigen presentation and increase cytotoxic killing of tumor cells.^34^ Despite a promising 20–40% response rate in pilot studies with human leukocyte IFN (a mixture of IFN-α subtypes), subsequent monotherapy studies in advanced breast cancer patients were unsuccessful.^54^ There is one ongoing clinical trial using IFN-α with pembrolizumab and a therapeutic breast cancer vaccine (NCT03328026) in metastatic breast cancer. Combination therapy with low-dose IFN-β, IL-2 and tamoxifen was well tolerated, had immunostimulatory effects, a 46% response rate and a survival benefit in advanced breast cancer.^55^

Therapeutic strategies aimed at restoring type I IFNs through Stimulator of Interferon Genes (STING) signaling are being investigated in human malignancies.^56^ Intratumoral injection of the STING agonist, ADU-S100/MIW815, has been demonstrated to inhibit the growth of breast tumors in mice.^57^ A clinical trial investigating MIW815 combined with ipilimumab(NCT02675439) as well as a trial with MIW815 combined with spartalizumab (anti-PD-1; NCT03172936) are currently ongoing in advanced solid tumors.

### Antigen presentation

Recognition of antigens on the surface of tumors or presentation by APCs results in the generation of antigen-specific T-cell responses and potentially T-cell-mediated lysis. Common breast tumor-associated antigens (TAAs) include HER2, MUC1, CEA, NY-ESO-1, MAGE, brachyury, cMET, and mesothelin. Priming T-cells through vaccination to recognize tumor antigen and to eliminate cancer cells could prevent the development of metastasis. Vaccines elicit T-cells or B-cells that produce antibodies directed against immunogenic proteins. Although vaccine-induced antibodies specific for proteins such as growth factor receptors may have an impact on cancer cell signaling and limit tumor growth, CD8^+^ and T-helper 1 (Th1) cells secreting inflammatory cytokines are needed for tumor destruction.^58^ The administration of vaccine adjuvants like granulocyte-macrophage colony-stimulating factor (GM-CSF), IL-2 or T-cell costimulatory molecules may help to augment the immune response, including the recruitment of more DCs, CD8^+^, and Th1 cells to the TME.

Transgenic mouse models of breast cancer show that vaccination against multiple tumor antigens is superior to immunizing against a single protein.^59^ Vaccination against MUC1 and CEA results in generation of T-cell responses to a cascade antigen called brachyury.^60^ Brachyury is a transcription factor associated with tumor plasticity^61^ and is overexpressed in breast cancer, particularly TNBC. High brachyury expression is associated with therapeutic resistance and a poor prognosis.^61^ Vaccines targeting the tumor plasticity autocrine loop (Fig. 2) and breast cancer stem cells are under development.^62,63^

As effective antigen combinations are being evaluated, new methods of antigen delivery to enhance T-cell immunity are also advancing in the clinic. Most novel approaches have focused on improving “signal 1” (antigen recognition) or “signal 2” (co-stimulation of) T-cells. One method to improve antigen recognition is to identify tumor mutations seen by the immune system as “foreign.”^64^ However, with only 3.2% of breast cancers containing the number of nonsynonymous mutations that allow for neoantigen formation, this approach is limited.^65^

Trastuzumab is a monoclonal antibody that is the backbone of almost all HER2+ breast cancer treatment regimens. Although antibody blocking of cell signaling through the HER2 tyrosine kinase has long been considered to be the major mechanism of action, recent evidence indicates that the generation of adaptive immunity plays an important role in the clinical efficacy of trastuzumab. In vivo trastuzumab binds to HER2 and activates NK cells, increases HER2 uptake and processing by DCs, and enhances the generation of CD8^+^ T cells (e.g., ADCC).^66,67^ Trastuzumab treatment results in the development of T-cell and antibody immunity directed against HER2; in essence, trastuzumab binding to HER2 on breast cancer cells acts as a vaccine.^68,69^ In a neoadjuvant trastuzumab trial, the development of high levels of HER2-specific Th1 cells independently associates with pCR at the time of definitive surgery.^70^ Furthermore, trastuzumab treatment significantly increases the number of Tbet+ (a marker for Th1 and CD8^+^) T-cells infiltrating the tumor, and an increase in relapse-free survival is noted in those patients who have Tbet + TIL induced.^71^ A recent trial with trastuzumab plus the HER2-targeted vaccine nelipepimut-S and GM-CSF suggests clinical efficacy in HER2+ patients and, interestingly, a significant improvement in disease-free survival in patients with TNBC,^72^ reinforcing the importance of the off-target immune effects of HER2-targeted antibodies for disease control.

### Toll-like receptors

Antitumor innate immune responses are in part regulated by TLRs, RIG-I–like receptors (RLRs), and the STING signaling pathway.^56,73^ Emerging data indicate that targeting TLRs, RLRs, and STING signaling may be a promising approach in the treatment of cancer, either alone or in combination with other immunotherapy agents. TLRs are expressed on both immune cells and tumor cells. Activation of specific TLRs on tumor cells results in immune evasion.^73^ TLR activation, conversely, also stimulates antigen presentation, DC maturation, and priming of CD8^+^ T-cells. A TLR7 agonist has been shown to be synergistic with other treatment modalities in a mouse model of breast cancer.^74^ The combination of pembrolizumab and intratumoral SD-101 is being investigated in the I-SPY2 neoadjuvant clinical trial for women with early stage HER2-negative breast cancer (NCT01042379).

### Innate immunity

The innate immune system includes granulocytes (neutrophils, eosinophils, and basophils), DCs, NK cells, MDSCs, and macrophages. As illustrated in Fig. 2, this cellular network plays a vital role in antitumor immunity through direct tumor killing as well as initiating, supporting and skewing the adaptive immune response through secreted cytokines.

### Dendritic cells

DCs are a critical component of antitumor immunity and are the most efficient APCs. DCs play a large role in antigen-specific (cancer) immune tolerance. DCs are present in peripheral tissues in the immature form and mature with the assistance of proinflammatory cytokines such as IL-1, IL-6, IFN-γ, and TNF-α, as well as in response to damage-associated molecular pattern (DAMP) signals.^75^ Immature DCs are not as efficient as mature DCs, and accumulation of immature DCs induced by tumor-produced granulocytes leads to decreased immune surveillance.^76^ Mature DCs infiltrate tumors, stimulate CD8^+^ T-cells, increase antigen presentation and assist with T-cell expansion.^77,78^ There are various methods to help augment the conversion to mature DCs, including the supplemental use of GM-CSF, cytokines and the administration of DC-based vaccines.

Single-agent DC vaccines enhanced for antigen presentation (i.e., HER2, MUC1) have been evaluated in the preclinical and clinical settings. Promising preclinical data have not translated to clinical benefit. However, therapeutic strategies aimed at increasing the abundance of mature DCs in the TME may increase responses to anti-PD-1 therapy.^77^ There are multiple ongoing trials involving autologous DC vaccines in combination with various other treatment modalities.

### Natural killer cells

NK cells are innate lymphocytes that recognize and kill tumor targets directly or upon CD16 engagement on antibody-bound cells, triggering the release of cytotoxic granules, chemokines, and proinflammatory cytokines.^79^ The strongest data supporting the role of NK cells in breast cancer are in HER2+ breast cancer. In preclinical models, NK cells are critical in the antitumor responses mediated by HER2-targeting antibodies.^77,78^ Trastuzumab induces ADCC, which leads to antigen release, cross-presentation by DCs, and increased NK cell activation and migration. Baseline tumor-infiltrating NK cells in primary HER2+ breast cancers are a predictive biomarker for pCR to anti-HER2 antibody therapy.^80^ In breast cancer patients, increased NK infiltration in the TME has been observed upon treatment with HER2-targeting agents, supporting the notion that NK cells are important contributors to the antitumor activity observed with currently approved HER2-targeted therapies.^81^ Multiple ongoing clinical trials are evaluating the impact of NK-directed treatment on the efficacy of HER2-targeting agents (NCT02627274; NCT03319459).

### Myeloid-derived suppressor cells

MDSCs are a heterogeneous population of cells that inhibit T-cell function. MDSCs increase with stage and metastasis,^82^ and may serve as potential target for amplifying host immunity.^83–86^ There are no known selective MDSC inhibitors in development; however, many existing drugs have effects on MDSCs. For example, DNA methyl transferase inhibitors and histone deacetylase (HDAC) inhibitors reduce systemic and intratumoral MDSCs, resulting in augmentation of immunotherapy over time.^83,87^ Chemotherapeutic agents that suppress/deplete MDSC and may augment the impact of immunotherapy include gemcitabine, doxorubicin, and 5-fluorouracil.^85,88,89^ Romidepsin, an HDAC inhibitor, is being evaluated in combination with cisplatin and nivolumab in TNBC (NCT02393794). Another agent that decreases MDSCs and enhances anti-PD-l efficacy preclinically^90^ is IPI-549 (IPI-145 or duvelisib). IPI-549 is an inhibitor of PI3Kδ and PI3Kγ isoforms and is being evaluated with nivolumab in solid tumors (NCT02637531).

### Tumor-associated macrophages

In breast cancer, the dominant TAM phenotype is tumor promoting (also known as M2 macrophages).^91^ TAMs promote tumor growth, angiogenesis, invasion, metastasis, as well as resistance to therapy.^44,92^ The underlying mechanisms include inhibition of CD8^+^ T cells, degradation of extracellular matrix, stimulation of angiogenesis and inhibition of phagocytosis.^92^ In a meta-analysis including over 2000 breast cancer patients, high TAM density in the primary tumor predicted worse patient prognosis.^92,93^ Furthermore, expression of macrophage colony-stimulating factor (CSF1) and its receptor (CSF1R) on TAMs has been correlated with poor prognosis in breast cancer.^94^

Preclinical studies of TAM-targeted therapies are primarily aimed at inhibiting macrophage recruitment, survival, and tumor-promoting activity in tumors, but the most potent antitumor strategy could be skewing tumor-promoting M2 TAMs to tumor-suppressing, immunostimulatory M1 macrophages. TAM-targeting strategies (e.g. TAM depletion/reprogramming/targeting functional molecules) have been proposed to enhance the efficacy of ICB.^95^ Targeting TAMs with CSF1 inhibitors leads to decreased TAM infiltration, reduced tumor growth, reduced metastases and prolonged survival in a breast cancer xenograft mouse model.^92^ There are multiple ongoing trials involving CSF1/CSF1R-targeting agents in breast cancer as monotherapy (NCT02265536) and in combination with chemotherapies (NCT01596751; NCT02435680). Macrophages can also be therapeutically targeted by inhibition of Tie2 kinase on Tie2-^High^/VEFG-^High^ TAMs. This subpopulation of TAMs form microanatomic structures that act as sites for cancer cell dissemination when in direct contact with endothelial cells. Furthermore, tumor cells can express invasive isoforms of the Mena protein, which creates a tumor microenvironment of metastasis (TMEM).^96^ Higher TMEM density is associated with higher risk of distant recurrence in localized breast cancer.^97,98^ Neoadjuvant chemotherapy can lead to a higher TMEM density,^98^ suggesting a previously unrecognized mechanism of resistance to cytotoxic therapy.^99^ Rebastinib is a Tie2 kinase inhibitor that targets these TAMs and reduces hematogenous seeding at intravasation sites.^100^ Rebastinib is being evaluated in combination with paclitaxel and eribulin mesylate (NCT02824575).

### Intrinsic vs acquired resistance to immunotherapy in breast cancer

The low response rates achieved with single-agent ICB in breast cancer reflects intrinsic or innate resistance,^12,13^ and understanding the mechanistic basis of resistance will inform future therapeutic strategies, including the development of rational combinations. Presently, little is known about acquired resistance to ICB in breast cancer. The largest clinical trials of anti-PD-1/L1 antibodies suggest that duration of response may be shorter on average that in other cancers, but these trials have not characterized the clinical or molecular features of resistant disease. In cohorts of patients with melanoma and non-small cell lung cancer (NSCLC), acquired resistance often presents as progression at a limited number of sites, implying that local immune or tumor heterogeneity underlies this phenomenon.^101,102^ Other mechanisms that may contribute to acquired resistance include loss of T-cell function, loss of T-cell tumor recognition and resistance to the effects of IFNγ produced by T-cells.^103^

Adaptive changes in immune checkpoint expression have also been identified as a potential mediator of acquired resistance to ICB. For example, upregulation of TIM-3 on T-cells has been observed in mouse models as well as in human NSCLC progressing after initial brief responses to ICB.^104^ Chronic IFN signaling resulting in multigenic adaptive changes in expression of T-cell inhibitory ligands is one mechanism shown to underlie the emergence of such resistance.^105^

Understanding the ‘cold’ immune microenvironment is the subject of intensive ongoing research. The absence of TILs in the microenvironment could point to defects in the adaptive immune cycle. First, it could suggest that too few neoantigens are present in order to signal a non-self. Strategies to overcome defects in this area include tumor foreignization, activation of endogenous retroviral sequences,^106^ or direct agonists of innate immunity (e.g. STING agonists). Secondly, it could suggest defects in the antigen presentation process. For instance, genomic loss of β_2_-microglobulin, a prerequisite component of MHC-1/peptide complexes, has been identified in relapsed melanoma and microsatellite high colorectal tumors after initial response to immunotherapy.^107,108^ Thirdly, a poor TIL presence could indicate suppressive signals in the breast cancer microenvironment that exclude T-cells and other effector cells^109^ from intratumoral regions. Examples include TGFβ and B7-H4 expression by breast tumor cells, each of which could be targeted by the immunotherapeutic strategies described above.

## Conclusion

In this review, we have highlighted only a few of the preclinical approaches and ongoing clinical trials in breast immuno-oncology. Currently, there are hundreds of ongoing clinical trials in the field of breast immuno-oncology with many of these trials combining immuno-oncology agents and/or standard of care regimens.^110^ Similar to current standard of care breast cancer treatment, a single approach for all breast cancers will likely not work. Breast cancers are complex with different subtypes not only harboring varying expression of targetable receptors (i.e., ER, PR, HER2) but also varying expression of PD-L1 and TAAs. Tumor mutational burden varies significantly by breast cancer subtype. Inflammatory cytokines also appear to have varying expression by breast cancer subtype.

Breast cancers are not cold tumors devoid of immune infiltration. Rather, immune cells are present in the tumor and the TME, but the environment is often immunosuppressive. Understanding the role of the innate and adaptive immune systems in breast cancer may provide guidance to improving the antitumor immune response by (1) intentionally expanding effector T-cells, NK cells and immunostimulatory DCs, (2) improving antigen presentation, and (3) decreasing inhibitory cytokines, tumor-associated M2 macrophages, MDSCs and Treg cells. These interventions, in turn, can shift the balance in the TME, and make breast cancers more responsive to immunotherapy. Perhaps, if we build it (an immuno-permissive TME) through these multiple approaches, they (TILs) will come.

## Data Availability

Source data for Figs. 1–3 and Tables 1 and 2 are provided in the manuscript. Clinicaltrials.gov was used to identify ongoing clinical trials mentioned in the manuscript. No new datasets were generated or analyzed for this report.
